# Stable, three degree-of-freedom myoelectric prosthetic control via chronic bipolar intramuscular electrodes: a case study

**DOI:** 10.1186/s12984-019-0607-8

**Published:** 2019-11-21

**Authors:** Hendrik Adriaan Dewald, Platon Lukyanenko, Joris M. Lambrecht, James Robert Anderson, Dustin J. Tyler, Robert F. Kirsch, Matthew R. Williams

**Affiliations:** 1 Department of Biomedical Engineering, Case Western Reserve University, 10,900 Euclid Avenue, Cleveland, OH 44106–1712 USA; 2 Cleveland FES Center, Louis Stokes Cleveland Veterans Affairs Medical Center, 10701 East Boulevard, B-E210, Cleveland, OH 44106-1702 USA; 3 APT Center, Louis Stokes Cleveland Veterans Affairs Medical Center, 10701 East Blvd., Mail Stop 151 W/APT, Cleveland, OH 44106-1702 USA; 4 UH Cleveland Medical Center, 11100 Euclid Ave, Cleveland, OH 44106 USA

**Keywords:** Transradial amputation, Implanted intramuscular electrodes, Myoelectric control, Neuroprosthetics, Prosthetic limbs

## Abstract

**Background:**

Modern prosthetic hands are typically controlled using skin surface electromyographic signals (EMG) from remaining muscles in the residual limb. However, surface electrode performance is limited by changes in skin impedance over time, day-to-day variations in electrode placement, and relative motion between the electrodes and underlying muscles during movement: these limitations require frequent retraining of controllers. In the presented study, we used chronically implanted intramuscular electrodes to minimize these effects and thus create a more robust prosthetic controller.

**Methods:**

A study participant with a transradial amputation was chronically implanted with 8 intramuscular EMG electrodes. A K Nearest Neighbor (KNN) regression velocity controller was trained to predict intended joint movement direction using EMG data collected during a single training session. The resulting KNN was evaluated over 12 weeks and in multiple arm posture configurations, with the participant controlling a 3 Degree-of-Freedom (DOF) virtual reality (VR) hand to match target VR hand postures. The performance of this EMG-based controller was compared to a position-based controller that used movement measured from the participant’s opposite (intact) hand. Surface EMG was also collected for signal quality comparisons.

**Results:**

Signals from the implanted intramuscular electrodes exhibited less crosstalk between the various channels and had a higher Signal-to-Noise Ratio than surface electrode signals. The performance of the intramuscular EMG-based KNN controller in the VR control task showed no degradation over time, and was stable over the 6 different arm postures. Both the EMG-based KNN controller and the intact hand-based controller had 100% hand posture matching success rates, but the intact hand-based controller was slightly superior in regards to speed (trial time used) and directness of the VR hand control (path efficiency).

**Conclusions:**

Chronically implanted intramuscular electrodes provide negligible crosstalk, high SNR, and substantial VR control performance, including the ability to use a fixed controller over 12 weeks and under different arm positions. This approach can thus be a highly effective platform for advanced, multi-DOF prosthetic control.

## Background

One of the largest issues facing upper extremity prosthesis development is a high rate of abandonment, with roughly 41% of surveyed amputees ending use of modern electric prosthetic hands citing limited functional gain among their rationale [1]. For transradial amputees, this “modern electric prosthetic hand” typically refers to commercially available prostheses that utilize surface electromyography (surface EMG) for sequential control of 2 or fewer Degrees-of-Freedom (DOFs) [2]. However, despite this high level of rejection, electric hands (as opposed to body powered or cosmetic hands) still “sparked the greatest interest for future use” in surveyed amputees, with the two largest concerns for their ongoing development being comfort and function [3]. Much of the prosthetics research of the past decade has thus focused on improving mechanical hand performance, with newer and more capable multi-DOF prosthetic devices (such as the DEKA [4, 5] and MPL [6, 7] arms, or hands such as the Michelangelo and iLimb [8]) providing greater levels of possible functional return compared to previous prostheses. However, the actual control of these devices is often rather limited, with some requiring additional external information (such as from an accelerometer on the foot [9]) or frequent “retraining” [10, 11] to function appropriately.

The EMG signal provides a near-direct measure of movement intent via activation of relevant residual musculature [12], thus providing the basis of a natural and intuitive interface. However, commonly used surface EMG, while unobtrusive and noninvasive, can have serious drawbacks. Advanced multi-DOF controllers based on surface EMG signals require regular retraining, otherwise suffering a loss in performance due to environmental skin impedance changes (e.g., due to sweat), electrode placement variation over time, or electrode lift-off during movement [13]. Other potential concerns regarding surface EMG include channel crosstalk, lack of access to deeper residual muscles, and/or too few electrodes for the number of controllable DOFs desired [14].

In comparison to surface EMG, intramuscular techniques allow access to deeper musculature so as to improve throughput and performance [15], are not subject to changes in skin impedance or the surrounding environment, provide improved grip force control [16], and experience less crosstalk than surface electrodes [17]. Early work had suggested improved control when using temporary fine wire intramuscular electrodes versus surface electrodes [15, 18, 19], but results were mixed [20]. *Chronically implanted* intramuscular EMG electrodes have long been used in other patient populations, such as for the control of a hand neuroprosthesis for tetraplegia [21], and have demonstrated years of stability; recently, chronically implanted systems for use with amputees have emerged in the prosthetics research field, such as the Implantable Myoelectric Sensors (IMES) [22] and the Osseointegrated Human-Machine Gateway (OHMG) [16, 23, 24]. However, evaluations of the impact of such a system on multi-DOF hand controller stability, both over long periods of time and with the residual limb in various positions, have been limited.

The goal of this case study was to evaluate the potential effectiveness of chronically implanted intramuscular electrodes in enhancing multi-DOF prosthetic hand functionality through improved controller stability, both over time (months) and for a range of different arm positions.

## Methods

### Participant selection and surgery planning

The study participant was a 37-year-old adult male with a left-sided transradial amputation who previously used a myoelectric prosthetic hook. He suffered from no condition that was considered exclusionary, such as any higher than normal risk for infection or exhibition of significant phantom or residual limb pain. He was also made aware of the surgical risks, the ongoing need for percutaneous lead maintenance, the expectation of future X-rays, the contraindication for MRI, and the explantation procedures before providing his consent to enroll in the study. Prior to surgery, ultrasound was used to determine eight potential residual musculature targets for the intramuscular electrodes, along with potential alternative muscles, as listed in Table 1. This protocol was approved by the Louis Stokes Cleveland Department of Veterans Affairs Medical Center Institutional Review Board (IRB #16050-H37) and performed under an active Food and Drug Administration (FDA) Investigational Device Exemption (IDE), G110043.

**Table 1:** Muscle Selection. This table shows the original surgery plan, alternative site options, and the final selections. The electrodes were inserted in the numerical order in which they are shown, and the surgical time needed for implantation is indicated for each muscle

Electrode	1	2	3	4	5	6	7	8
Initial Plan	Pro T.	FCU	FDS	FPL	Sup.	ECRB	EDC	EPL
Alternatives		FCR	FDP			ECRL	EDMP	APL
Final Setup	Pro T.	FCR	FDS	FCU	Sup.	ECRL	EDC	ECU
Time Needed (min)	18	2	2	2	2	2	2	3

The acronyms are defined as follows: *Pro T.* Pronator Teres, *FCR* Flexor Carpi Radialis, *FCU* Flexor Carpi Ulnaris, *FDP* Flexor Digitorum Profundus, *FDS* Flexor Digitorum Superficialis, *FPL* Flexor Pollicis Longus, *ECRB* Extensor Carpi Radialis Brevis, *ECRL* Extensor Carpi Radialis Longus, *Sup.* Supinator, *EDC* Extensor Digitorum Communis, *EDMP* Extensor Digiti Minimi Proprius, *EPL* Extensor Pollicis Longus, and *APL* Abductor Pollicis Longus

### Intramuscular electrode insertion

**Fig. 1:**
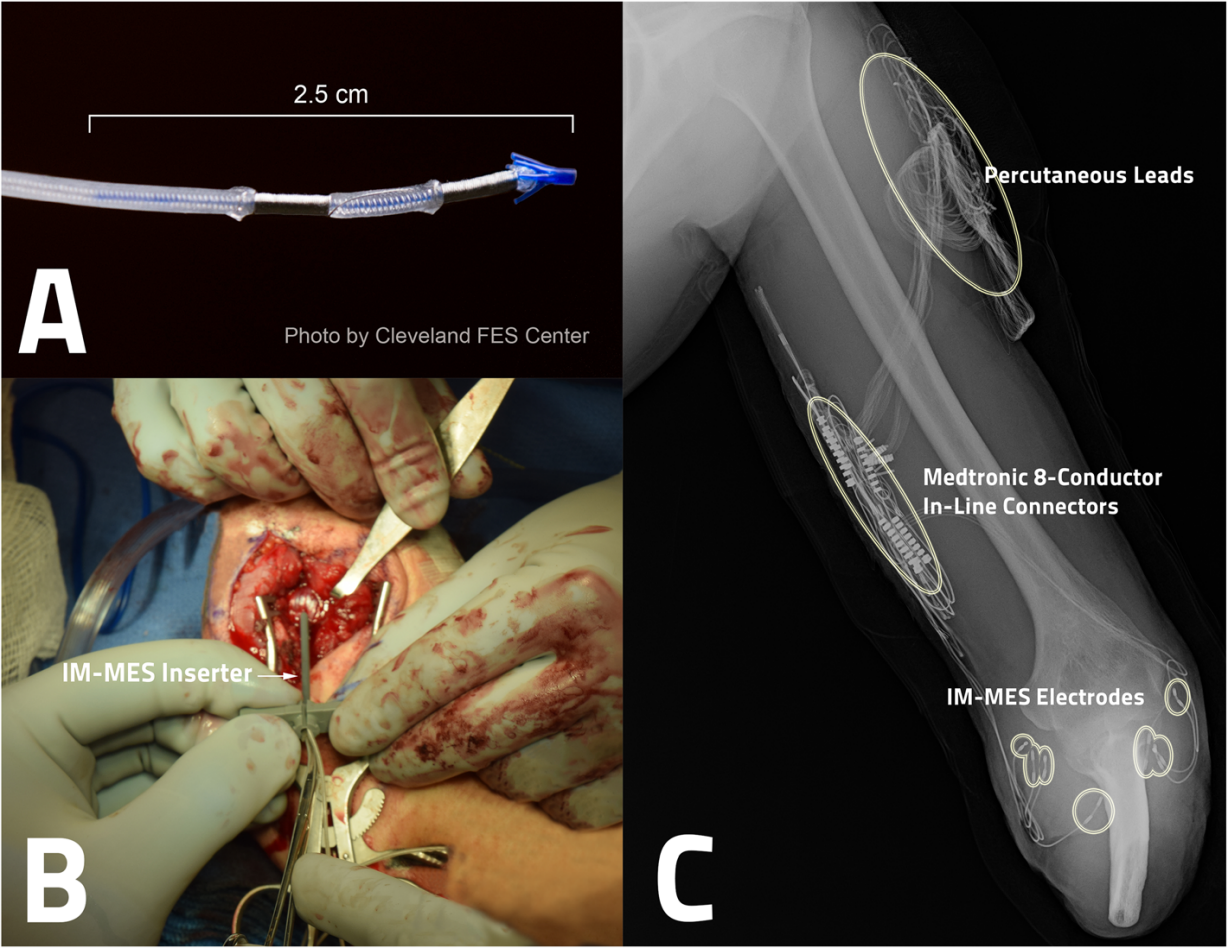
IM-MES Implantation. **a** One of the eight IM-MES electrodes. **b** One such electrode being surgically tunneled into a target muscle of the study participant’s residual limb using the IM-MES insertion tool. **c** X-ray showing the IM-MES electrodes, connectors, and leads following the successful procedure, as well as additional nerve cuff electrodes (unmarked) implanted in the same surgical procedure for use in a separate study

**Fig. 2:**
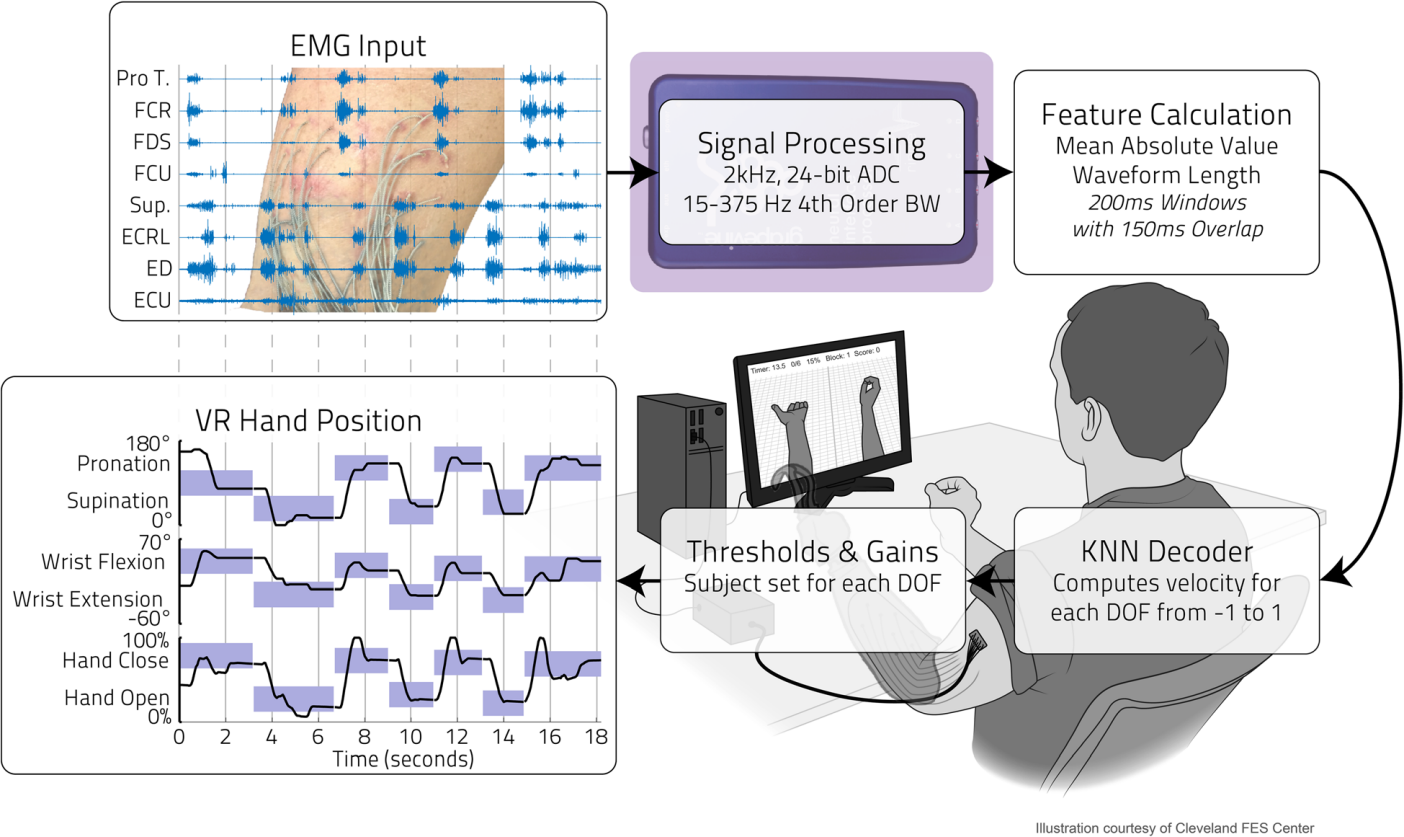
Real-time Control Flowchart. EMG data (top left) from the 8 implanted electrodes (with upper arm percutaneous lead exit sites shown behind) is collected by the Ripple Grapevine Neural Interface Processor (shown top center behind the signal processing text), which applies a Butterworth (BW) bandpass filter and digitally samples the signal (ADC: Analog-to-Digital Converter). Two features (Mean Absolute Value and Waveform Length) are then calculated from a 200 ms window of this EMG data every 50 ms and subsequently fed to a decoder. This regressive K Nearest Neighbor (KNN) controller computes a velocity vector for a virtual reality (VR) hand posture matching program (shown in the bottom right drawing). The gain (relationship between predicted and outputted virtual hand motion) and velocity threshold (below which all velocities are considered erroneous and set to zero) are then adjusted according to participant preferences. The resulting VR 3-DOF hand position is shown in the bottom left. The purple rectangles mark the target hand posture ranges for each DOF, with the black lines indicating the VR hand’s relative positions in those DOFs. The longer the purple rectangle, the more time the participant took to reach and keep that target hand posture for the required dwell time of 1 s

Eight bipolar IM-MES electrodes [25] were inserted surgically into the proximal origins of wrist and finger muscles of the study participant (Fig. 1). The leads of these intramuscular electrodes (two per electrode) were connected to implanted 8-conductor in-line connectors (Medtronic Model 37,081 low impedance extension kits, Medtronic, Minneapolis, MN), which then exited through the skin as tandem-wound open helix percutaneous leads (Fig. 2). As shown in Table 1, the presence of limited thumb musculature was pre-surgically indicated via Ultrasound. However, during surgery, different superficial wrist muscle sites were targeted to avoid the extensive dissection or blind insertion needed to access the proximal origins of said thumb musculature. The first electrode insertion required 18 min, but this decreased to as little as 2 min per electrode as the surgery progressed and the surgeons became more familiar with the insertion procedure. Each electrode was inserted through one of two 3 cm incisions. The leads connecting to the intramuscular electrodes were tunneled through the upper arm and routed through the skin near the shoulder. The study participant underwent a 3-week recovery period before any data sets were collected.

### EMG collection and processing

Electromyographic data (Fig. 2, top left panel) was sampled from the eight sites using the Ripple Grapevine Neural Interface Processor (NIP) system with an EMG front end for the intramuscular IM-MES setup and a Touchproof adaptor for the surface EMG setup (Ripple, Salt Lake City, UT). The signals were sampled at 2 kHz with 24-bit resolution after application of a 4th order Butterworth 15-375 Hz bandpass filter. A surface electrode with a gel adhesive (DIN EMG snap leads from Bio-Medical Instruments) was attached at the lateral epicondyle of the elbow as the recording reference. A custom-built Simulink model (The MathWorks, Natick, MA) acquired a 200 ms buffer of EMG activity from the Ripple Grapevine NIP every 50 ms over an Ethernet connection. Mean Absolute Value (MAV) and Waveform Length (WFL) features [26, 27] from each 200 ms buffer were then used as inputs for use by a controller (described later).

### Surface EMG setup

Surface electrodes were applied during one experimental session so as to provide a comparison for crosstalk and signal-to-noise ratio (SNR). Each channel consisted of a bipolar pair of electrode contacts of the same make as used for the previously mentioned recording reference, and eight channels were chosen to match the eight channels of the IM-MES setup. We found that the limited space on the limb precluded adequate muscle targeting, and so decided upon an untargeted surface electrode setup with the eight pairs evenly spaced around the circumference of the residual limb [11, 14, 28].

### Virtual reality setup

A virtual reality (VR) program was used to display two virtual hands (as in Fig. 2), both for collection of training data and for evaluation of the effectiveness of various hand controllers (to be described below). The VR environment was created using GameStudio (Conitec, La Mesa, CA) and has been used in prior studies [29–32]. The VR was managed by the same custom Simulink model responsible for collecting the EMG and kinematic data described above. A VR approach was used to quantitatively measure the speed and accuracy of control, with VR performance metrics shown to correlate to functional outcome metrics with a physical prosthesis [33]. For this case study, 3 DOFs of the virtual hand and forearm were controllable/adjustable: forearm supination/pronation (0 to 180° range of motion), wrist extension/flexion (− 60 to 70° range of motion), and hand open/close (0 to 100% range of motion).

### Intact hand comparison

Kinematic data (forearm pronation, wrist flexion, and index metacarpophalangeal (MCP) flexion angles) from the intact arm and hand were collected using an electrogoniometer and torsiometer setup (Biometrics Ltd., Ladysmith, VA). A Single Axis Goniometer was fastened between the index finger and the back of the hand to measure hand opening, a Twin Axis Goniometer was placed between the back of the hand and forearm to measure wrist flexion and extension, and a Single Axis Torsiometer was placed on the forearm to measure forearm pronation-supination. The analog signal outputs from the goniometer and torsiometer’s K800 Amplifier were sampled at 1 kHz with 16-bit resolution by the Ripple Grapevine NIP system using an Analog Input/Output (I/O) front end, and were buffered in the same fashion as the EMG recordings (with a mean calculated from a window of 200 ms and updated every 50 ms). The outputs were then calibrated to the ends of the subject’s volitional range of motion for each of the 3 DOFs of the intact hand. The resulting setup thus linearly mapped each of 3 DOFs of interest of the intact hand to its corresponding DOF in the VR hand (i.e., a Position Controller). This controller was used as an example of best-case performance against which to measure the IM-MES based controller described below.

### Data collection for training the machine learning controller

**Fig. 3:**
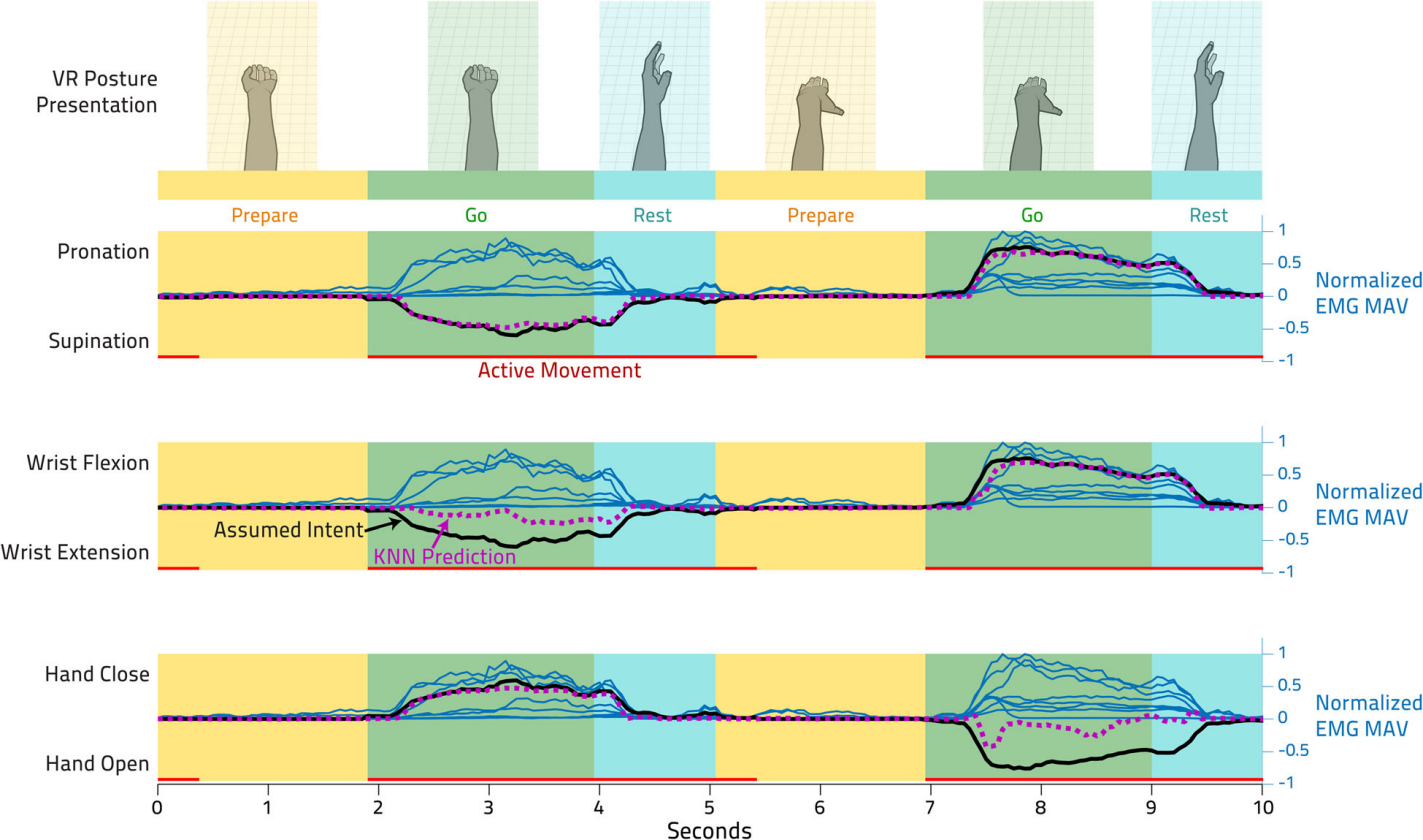
Data Processing for K Nearest Neighbor Training. Training data (two EMG features from each of the eight muscles) was collected during the presentation of target VR hand postures. Note that, for clarity, only the normalized Mean Absolute Value (MAV) features are shown here as the thin blue lines, one for each muscle. The target VR hand posture was initially presented to the participant during the ‘Prepare’ phase, shown as yellow rectangles. At the start of the ‘Go’ phase, shown in green, the participant continuously attempted to move their phantom hand to the presented hand posture. After 2 s, a rest posture was presented and the participant relaxed during a ‘Rest’ phase- these are shown in sky blue. Two 3 DOF target hand postures are presented in this illustration, requiring (1) simultaneous supination, wrist extension, and hand closing (shown above the leftmost ‘Prepare’ and ‘Go’ phases) and (2) simultaneous pronation, wrist flexion, and hand opening (shown above the subsequent ‘Prepare’ and ‘Go’ phases). The ‘Assumed Intent’ (thick black line) was computed by normalizing the movement needed to reach the target by the average of the rectified EMG of the sections marked ‘Active Movement’ by the red line. The dotted purple line represents how the regressive KNN trained from all collected training data ultimately predicted the 3 velocity signals from the EMG features

To collect training data for the K Nearest Neighbor (KNN) controller, the VR hands were shown mirroring one another in 27 randomly presented target hand postures. The 3 DOFs of each of these target hand postures were either at the end of their range of motion or at the neutral position, such that the 3 DOFs with 3 possible positions each resulted in the total of 27 possible configurations. For each hand posture, the participant was instructed to plan his movement for 2 s (‘Prepare’), attempt to perform the cued movement with both his intact and phantom hand for 2 s (‘Go’), and then relax for 1 s (‘Rest’), as shown in Fig. 3. Visual cues indicated the transitions between these three phases. Inspection of EMG recordings throughout these phases showed that the majority of activation was throughout the ‘Go’ phase, but some activity could regularly be seen continuing roughly 1.5 s into the following ‘Rest’ and ‘Prepare’ phases. The data used to train the KNN (marked in red as ‘Active Movement’ in Fig. 3) was thus defined as to comprise of the ‘Go’ phase plus the first 1.5 s of the ‘Rest’ phase. Each of the 27 hand postures was displayed 5 times, and the EMG data and target hand postures were recorded all throughout. This training data collection process took roughly 20 min, including breaks. The participant was instructed to contract at a low to moderate level, such that prolonged activity would not lead to fatigue during either the training data acquisition or the subsequent online (real-time) controller use. The participant wore his socket with the myoelectric prosthetic hook throughout all these sessions, though it was not active.

### Controller data processing

In the complete training dataset, each of the 16 feature dimensions (8 EMG channels, each with both MAV and WFL features, thus resulting in said 16 dimensions) was normalized to have a maximum value of 1. Then, each of the 27 hand postures was interpreted as a 3-dimensional movement vector so as to denote direction and magnitude for each DOF, formatted as [Pronation/Supination, Wrist Flexion/Extension, Hand Close/Open] (e.g. [1,0,0] would be full pronation and [− 1,1,0] would be full supination and wrist flexion). There was no way to independently measure the participant’s “intent”, so we computed an approximate ‘Assumed Intent’ by (1) normalizing the 3D movement vector at all time points in the ‘Active Movement’ period by the overall mean of the 8 EMG MAV signals, providing an estimate of overall intended effort under the rationale that magnitude of EMG activity is indicative of intended force [12], and (2) setting the 3D movement vector at all time points outside of the ‘Active Movement’ period to [0,0,0], under the assumption that any EMG activity during this period was either noise or erroneous behavior from the subject with no interpretable directional intent. The resulting ‘Assumed Intent’ constructed by this process is shown as a black line in Fig. 3. Each of the 16-dimensional EMG feature data points (again, from the 8 channels of both MAV and WFL) thus had a corresponding 3 DOF ‘Assumed Intent’ data point that served as the target for subsequent KNN training.

### K nearest neighbor controller

A simple nearest-neighbors regression was used to characterize the relationship between the previously described 16-dimensional EMG feature data and the 3-dimensional DOF velocity target data - i.e., to define the controller. During online VR hand posture matching tasks, all 16 EMG features were calculated continuously every 50 ms. At each of these points in time, the Euclidean (ordinary straight-line) distances between this 16-dimension EMG-based value and all 16-dimension EMG feature values of the training data were calculated. The closest 100 points were found, and the corresponding 3-dimensional target vectors of these 100 points were averaged and weighted by the inverses of their respective distances. This resulted in a single 3-dimensional vector at every time point that provided an estimate of user intent for each of the 3 DOFs between − 1 and 1. This 3 DOF vector was then used as a velocity input for the VR hand. This approach is known as a K Nearest Neighbor regression [34], where in this case the K is the 100 nearest points found and the regression is inverse-distance-weighted. This KNN approach was chosen for its characteristically short “training” time (as no weights or biases need to be calculated), its previous use in evaluating useful EMG features [27], and a short online controller delay (< 50 ms).

### Posture matching task

For assessing controller performance, the output of the KNN was used as the velocity command for the 3 DOFs of the left VR hand (the “virtual prosthesis” taking the place of the participant’s amputated limb) while the right VR hand displayed target postures. The velocity inputs had adjustable thresholds and gains per DOF to respectively reduce unintended movements and achieve the participant’s desired maximum speed. This was user subjective and could vary session to session, with thresholds typically around ±10% of the normalized velocity range and gains generally around 3, though occasionally getting as high as 10. This velocity signal is in percentage per second for the Hand Open/Close DOF and degrees per second for the remaining two DOFs.

The participant was instructed to match the target hand posture as quickly and accurately as possible and to hold that position until a success was indicated with an audible tone. Successful target acquisition required a position accuracy within ±15% of the target hand posture (relative to the range of motion for each DOF) and the ability to hold this posture for at least 1 s. The study participant had to match the posture under these conditions and within 30 s or the trial would be counted as unsuccessful. The study participant’s prosthetic hook was worn but not active during these hand posture matching tasks, as was the case during training data collection.

As a presumably best performance comparison, the participant also performed two hand posture matching sets using his intact hand’s movements to control the movements of the corresponding VR hand. In these sets, the position of the participant’s intact right hand was determined using the electrogoniometer setup previously described, and was directly mapped to the position of the VR right hand. The same hand posture matching tasks were then performed as with the KNN, but with the VR left hand displaying the target postures instead.

During each session, 80 target hand postures were sequentially and randomly generated, such that each target posture differed from the previous target posture by at least 30% of the range of motion for each DOF, and was at least 15% of the range of motion from the maximum and minimum (such that all targets could be overshot in all DOFs). The 80 targets were split into 5 blocks of 16 targets, with a participant-determined rest between blocks to prevent fatigue (though little, if any, was ever reported).

### Controller stability over time

Six experimental sessions were performed across a 12-week period to evaluate the long-term, between-session control stability (the “temporal stability”) of the KNN-based controller. The exact KNN parameters established in the initial training session were used in all of these subsequent sessions, and its performance over this 12-week period was quantified (Fig. 5). In the first session (the day the KNN was initially trained), two sessions with 80 targets each were performed. During subsequent visits, only one 80 target session was performed. As noted above, the KNN controller was maintained constant across all of these sessions – no additional training was done. Posture matching using the opposite intact hand-based controller was also performed at two time points (Week 0 and Week 2) to serve as a reference for a best-case controller scenario. Note that these were performed prior to the IM-MES based KNN sessions.

### Controller stability with arm position changes

The same KNN from the temporal stability evaluation previously described was also used when evaluating the controller stability during changes in residual limb’s position (the “postural stability”), with the residual limb (with the hook prosthesis on the arm) held in the various positions shown in Fig. 6. As compared to the previous sessions, a block of 16 target hand postures was presented in each of five different non-rest limb positions, wherein every other target hand posture was the neutral posture (the mean of all ranges of motion) and between these neutral postures was a randomly selected target posture near the minimum/maximum in all 3 DOFs (10% or 90%, to prevent overshoot). Between each of these blocks/limb positions was a short, participant determined rest period to prevent fatigue, which was more prevalent due to the required arm positioning. The ‘Rest’ condition trials were performed with the arm supported at the side and consisted of the five full blocks of the 16 hand target postures (80 total). The weighted condition consisted of the participant holding a cylinder weighing 650 g with his hook prosthesis. This was done in a single session.

### Posture matching metrics

Online performance was quantified by three metrics. The first was *Posture Matching Success Rate*, which was simply the percentage of target hand postures successfully matched under the previously listed criteria of ±15% range per DOF for the minimum of 1 s and within the 30 s trial duration. The second was *Path Efficiency* [35], which is defined as a measure of the straightness of the cursor path to the target and is computed by dividing the straight-line (or minimum) distance by the actual distance traveled in 3D space. Last was *Trial Time Used*, which was simply how many seconds it took to reach and keep the target hand posture as to mark as a success (which includes the 1 s dwell time requirement).

### Statistical analysis

To evaluate temporal stability (the stability of the KNN controller after multiple sessions), regression models for *Trial Time Used* and *Path Efficiency* versus *Controller* (KNN controller or intact hand-based controller) and *Weeks* (since the initial KNN training) were used, with interaction terms determined by ANOVA. If the resulting error term distribution was found to be non-normal, appropriate variable transformations were undertaken. Postural stability was evaluated by *Trial Time Used* and *Path Efficiency* through application of Kruskal Wallis tests for equality across the multiple residual limb configurations. EMG channel crosstalk comparison was done using a Box’s M Test to check the equality of two covariance matrices. EMG SNRs were calculated using an automated algorithm designed to estimate background noise, SNR, and duty cycle from EMG collected during cyclic contractions [36]. This approach was used on the repetitive training data collected using both Surface and IM-MES electrodes as a means of quantifying signal quality, with the resulting SNRs compared using a Wilcoxon Rank-Sum Test. For all following figures, we report the sample size, all data exclusions (if any), and all data manipulations of the study.

## Results

**Fig. 4:**
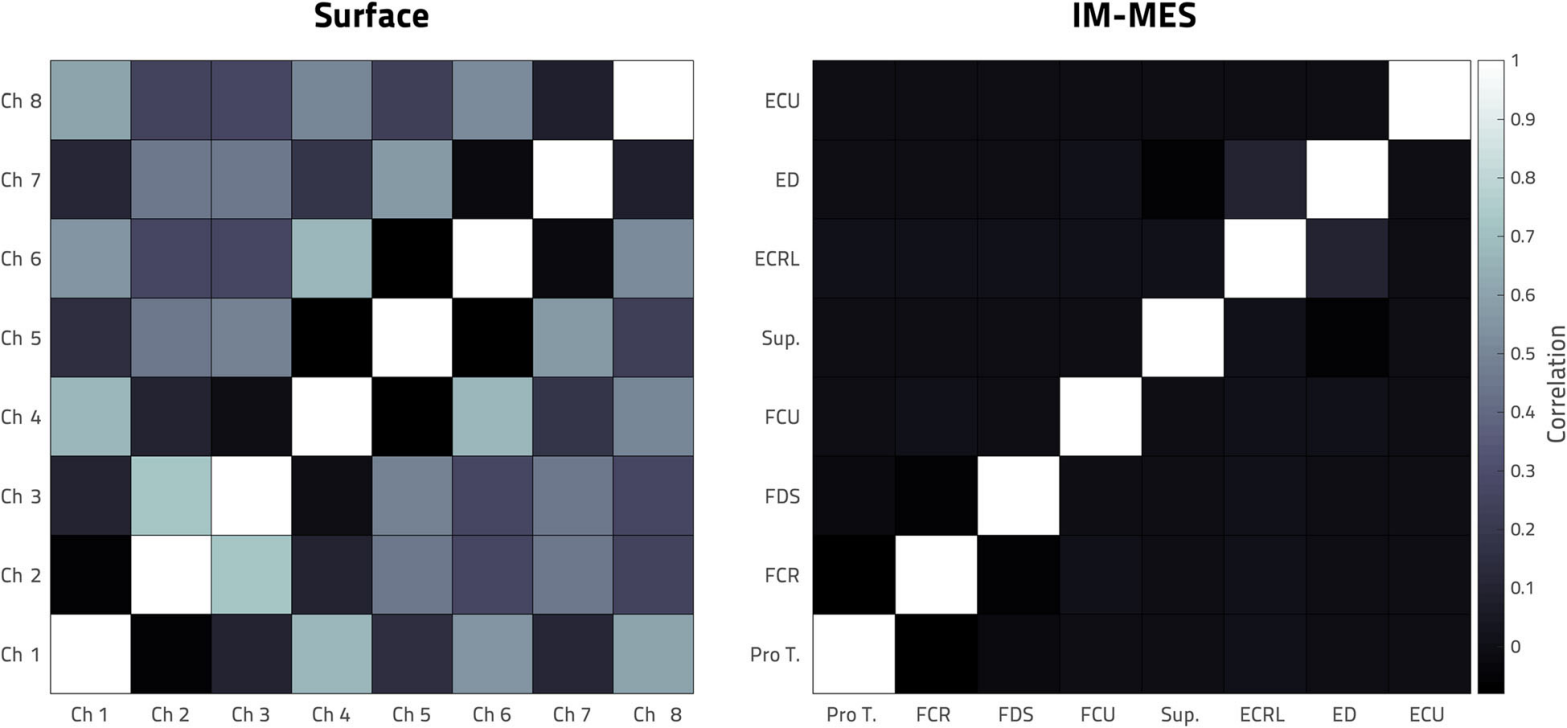
EMG Crosstalk. Signal cross-correlation is displayed here using a heat map. The brighter the square, the more correlation between the two channels. A case with no crosstalk between channels would be represented by black (representing 0) everywhere aside from a diagonal white line indicating each channel’s correlation with itself. The cross-correlations computed from the participant’s chronically implanted IM-MES setup are displayed on the right, while the cross-correlations from the 8-channel ring of surface electrodes on the same residual limb are displayed on the left

Cross-correlation was used to quantify the crosstalk between each of the bipolar intramuscular electrodes, as well as between each pair of surface electrodes. Figure 4 presents these cross-correlations in the form of a heat map, with a brighter colored square representing higher crosstalk between channels. It can be seen in the figure that the IM-MES electrode recordings exhibited very little crosstalk (average correlation between different electrodes was − 0.0024), while the surface electrodes exhibited clear crosstalk (average correlation between different electrodes was − 0.0842). The correlation matrices (i.e. crosstalk) of the IM-MES electrodes and the 8 surface EMG channels was found to be significantly different (p ≪ 0.001) using a Box’s M test.

Surface SNR was calculated from the training data of the single session where it was collected along with that of the IM-MES. IM-MES SNR was calculated from this same session as well as one of the subsequent sessions (“Week 0”). The average (across all 8 channels) surface SNR was found to be 25 ± 4 dB, while the average IM-MES SNR was found to be 34 ± 8 dB. Thus, the IM-MES signal magnitude is roughly three times greater relative to the noise than that of the surface setup. A Wilcoxon Rank-Sum test found the two significantly different with a *P* value of 0.014.

The singular KNN (“Week 0” KNN) used for all shown posture matching results was trained from IM-MES data collected in a single session on the same day as its first online evaluation. The “offline” performance of this KNN in predicting “Assumed Intent” from the 8 IM-MES signals of the training data, as measured by Variance Accounted For using a leave-one-out approach [34], was found to be 93.7% for forearm pronation/supination, 88.8% for wrist flexion/extension, and 63.1% for hand close/open.

### Temporal stability

For *Posture Matching Success Rate*, all sessions using either the “KNN” or “Intact Hand” controller had a 100% success rate under the requirements of the task, i.e., 1 s dwell time, 30s timeout, and ± 15% target requirement.

**Fig. 5:**
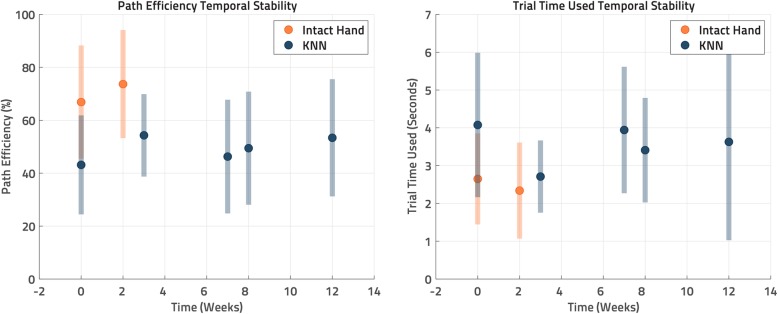
Posture Matching Performance Over Time. The same KNN constructed from data collected at the “Week 0” KNN mark was used during posture matching evaluation in every session. Each data point is an average of 80 target hand posture trials from one session with the exception of the first KNN session which was 160 trials from two sessions, one at the beginning and one at the end of the day. The error bars represent the standard deviation of each day’s collected trials. The greater the *Path Efficiency* and less the *Trial Time Used*, the better the performance

Figure 5 shows the *Path Efficiency* and *Trial Time Used* over the 12-week testing period, both for the IM-MES based KNN controller (blue points) and for the intact hand-based controller (orange points).

For *Path Efficiency*, the “Intact Hand”-based controller, as expected, was found to perform better than IM-MES based “KNN”, with the regression C_f_ term showing that the “Intact Hand” had a 21.8% advantage at Week 0. The regression also showed that the *Path Efficiency* of both “Intact Hand” and “KNN” controllers improved only slightly over time by 3.4% (C_w0_) and 0.6% (C_w1_) per week respectively Table 2.

**Table 2:** *Path Efficiency* Regression. A linear regression was performed relating *Path Efficiency* to both the controller (“Intact Hand” vs “KNN”) and an interaction between the controller and time in weeks, which was suggested by an ANOVA to be the best linear regression model. This regression shows better performance in *Path Efficiency* from the “Intact Hand” controller compared to the “KNN” controller, but there was no degradation in performance by either over time, as indicated by the interaction term coefficients

Model	Path Efficiency = (Intercept) + (C_f_)Controller + (C_w0/w1_)Week		
	Coefficient Estimate	Std. Error	*p*
(Intercept)	66.9	2.3	≪0.001
ControllerCoefficient C_f_ 0-Intact, 1-KNN	−21.8	2.7	≪0.001
Controller:Week C_w0_ (Intact)	3.4	1.6	0.03
Controller:Week C_w1_ (KNN)	0.6	0.2	0.002

Multiple R-squared: 0.196, Adjusted R-squared: 0.193

For *Trial Time Used*, a variable transformation was performed to remedy nonnormality of error terms and their impact on the regression model: the reciprocal of *Trial Time Used* was taken [37]. “Intact Hand” was found to allow faster posture matching than “KNN”, with Week 0 showing a 0.85 s advantage (from C_f_) for “Intact Hand”. Both coefficients for the interaction terms with week (C_w0_ and C_w1_) indicated that the *Trial Time Used* only decreased over the 12-week period in both controllers Table 3.

**Table 3:** *Trial Time Used* Regression. To provide a normal error term distribution, the *Trial Time Used* values underwent a reciprocal transformation. A linear regression was then performed relating *Trial Time Used* to both the controller (“Intact Hand” vs “KNN”) and an interaction between the controller and time in weeks, which was suggested by an ANOVA to be the best linear regression model. The “Intact Hand” controller performed better in *Trial Time Used* compared to the “KNN” controller, but there was no performance degradation over time in either, as indicated by the interaction term coefficients

Model	1/Time Used = (Intercept) + (C_f_)Controller + (C_w0/w1_)Week		
	Coefficient Estimate	Std. Error	*p*
(Intercept)	0.426	0.01	≪0.001
ControllerCoefficient C_f_ 0-Intact, 1-KNN	−0.113	0.02	≪0.001
Controller:Week C_w0_ (Intact)	0.031	0.01	0.001
Controller:Week C_w1_ (KNN)	0.003	0.001	0.04

Multiple R-squared: 0.202, Adjusted R-squared: 0.199

### Postural stability

**Fig. 6:**
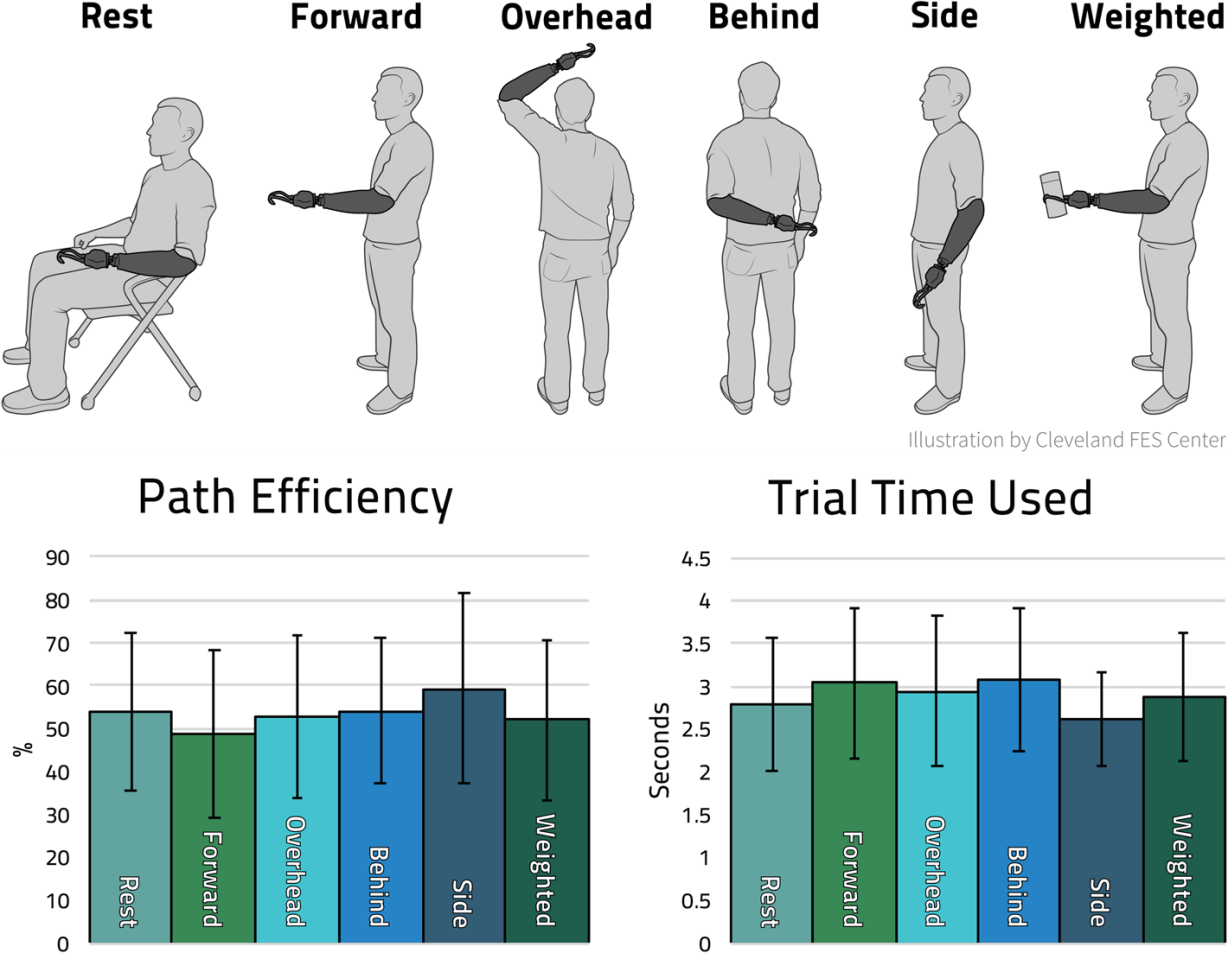
VR Posture Matching Performance During Various Arm Positions. The same single “Week 0” KNN used throughout all of Fig. 4 was also tested in a variety of arm postures shown in the illustrations above the bar plots. The weight applied in the last case was a 650 g cylinder at the end of his prosthetic hook. The ‘Rest’ condition was a full 80 target hand posture session, while the other conditions were 16 each to avoid participant fatigue. No significant difference was found between arm postures for either performance metric

Positioning the arm in different postures had no observable impact on the performance of the IM-MES based KNN controller. The *Posture Matching Success Rate* was 100% across all six arm postures and all sessions. And as indicated in Fig. 6, the performance of commanding the VR hand (as summarized by *Path Efficiency* and *Trial Time Used*) was essentially identical in all six arm postures. A Kruskal Wallis Test for Equality demonstrated no statistically significant differences in *Path Efficiency* (*p* = 0.77) or *Trial Time Used* (*p* = 0.58) between any of the six postures of the residual limb was placed in.

## Discussion

### Summary

This case study demonstrates that an implanted electrode setup reduces signal crosstalk, improves signal quality, and allows for robust postural and temporal stability. The participant was able to use 8 IM-MES signals from the residual limb to control 3 DOF motions of a virtual prosthesis with high performance. Specifically, hand posture matching had a 100% success rate over 12 weeks of testing and for six different residual arm postures. And the metrics of continuous control performance (Path Efficiency and Time to Target) using IM-MES signals with a KNN based controller were only slightly lower than those seen when the participant controlled the virtual prosthesis using the motions of his intact hand (a best-case controller). Furthermore, this high performance was provided by a **single**, fixed KNN controller that was trained at the beginning of the presented study and held constant (no retraining) for the entire 12-week duration - with no degradation in performance over time. The IM-MES based KNN controller evaluated allowed for accurate and precise movements within moments of activation and was capable of independent, proportional, and time-stable multi-DOF control through the intuitive and natural interface of EMG. This represents a significant improvement over the control interfaces used in most commercially available prosthetic devices.

### Performance of the implanted system

The implanted EMG system in this presented study provided a stable signal pickup regardless of the limb posture. Studies using surface electrodes for myoelectric control report difficulty in determining intent when arm posture changes [38], but the implanted electrodes are positionally stable in the muscle and therefore offer EMG signals more robust to movement. Because most prostheses use electrodes fitted to sockets, many amputees note a decrease in prostheses functionality when used overhead or in weighted conditions, but the consistent performance of IM-MES control in these various arm postures indicate that this approach can mitigate the issues with socket fit and changes to fit with posture. Supplementary videos by the OHMG group [23] has also suggested similar stability using their chronically implanted setup with a transhumeral amputee, but it wasn’t validated quantitatively.

The implanted system also provided a stable recording platform for a regressive controller over a 12-week period, and the training data from Week 0 is still being used in separate studies to this day, more than a year later. In comparison, any sufficiently complex multi-DOF control that uses surface EMG suffers a considerable loss in accuracy over multiple sessions without retraining [11]. Our results corroborate the findings by the OHMG group, who also showed no degradation in VR performance of a 3DOF pattern recognition classifier when using 6 chronically implanted intramuscular electrodes after 3 months [23]. Prosthetic hand functional performance as defined by day-to-day performance and initialization/retraining difficulty would thus be improved considerably with implanted intramuscular EMG electrodes compared to a surface electrode setup.

### Controllable degrees of freedom

Hand opening and closing is very typical for myoelectric prostheses, and pronation-supination (“wrist rotation”) is also available. However, currently available commercial prosthetic hands (other than the DEKA hand) typically do not offer active wrist flexion/extension. This is frequently due to a limited number of simultaneously controllable DOFs and the greater importance of controlling hand opening/closing. An active wrist reduces the need for compensatory movements (i.e. shoulder circumduction) by allowing a more general positioning of the hand. This case study shows that simultaneous, independent control of wrist flexion/extension with pronation/supination and hand open/close is immediately feasible. In the future, other combinations of hand/forearm DOF (e.g., wrist ulnar/radial deviation, alternative grasp postures, etc.) may also be controllable using this same approach.

### Controller approach

Various methods have been employed in prosthetic research to determine user movement intent and to control either physical devices or VR representations. This present study made use of a simple KNN, with a K of 100, to provide smooth control without excessively long training or computation times. A rather simple training approach was also utilized as to provide a uniform data set of possible movements for training in a short time frame (20 min). Training of the KNN was thus quick and easy, but the KNN requires significantly more computation during real-time control than many other machine learning approaches, and this computational burden increases rapidly as the number of controlled DOF increases beyond 3. Other machine learning approaches should be used for higher DOF control, more dynamic training approaches could be designed, and KNN (or other) controllers that operate sequentially rather than simultaneously could be evaluated.

### Study limitations

This case study only involved one participant, who underwent an invasive procedure. While the surgery had no major issues, the percutaneous site necessitated daily maintenance and dressing [39]. Normal percutaneous site management included infrequent presentation of a sweat gland blockage. No major infection has occurred over the 36 months since the implant, but three blockage events were documented and resolved through expression and cleaning. Two of these blockage events including precautionary broad-spectrum oral antibiotics; however, neither involved infection.

Because the relationship between VR hand posture matching tests and clinical performance is not explicitly established, it is unclear if the difference between the intact hand-based controller approach and the IM-MES KNN controller approach are functionally significant. Furthermore, the intact hand-based controller was a position controller, while the IM-MES KNN controller approach used velocity control instead. The effect of learning is also unclear due to the infrequent and brief nature of the posture matching evaluations.

We did not directly compare the IM-MES controller against a similar surface electrode controller, though we do not expect this to change our basic conclusions regarding the stability and advantages of intramuscular electrodes. Surface EMG cannot selectively record from deeper muscles, must be placed for each use, and are prone to signal changes due to skin impedance changes and sweat. Thus, surface EMG-based controllers require more frequent retraining [10]. Also, while we did compare the IM-MES signals to 8 surface electrodes to verify the advantages of implanted electrodes regarding improved signal quality and reduced crosstalk, we employed a simple circumferential ring setup seen frequently in the literature [11, 14, 28] instead of specifically targeting comparable musculature. However, a targeted surface electrode comparison would still exhibit sufficient crosstalk due to the required proximity (< 3 cm) of the 8 electrode pairs so as to not alter our conclusion [40].

Lastly, the impact of a reduction in crosstalk on performance is also ambiguous. A simple untargeted surface electrode setup like that used in our study has been suggested by Farrell et al. as functionally equivalent to both a targeted surface electrode approach and a targeted intramuscular electrode approach for the purposes of a pattern recognition classifier [41]. While crosstalk is a considerable issue for direct control methods wherein antagonistic muscle pairs control individual DOFs, it is quite likely to be less disruptive, or even helpful, to pattern classifiers [28].

### Future work

This study assessed performance using a virtual posture matching evaluation. While virtual environment control metrics are correlated to functional outcome metrics [33] and more thorough VR functional tasks could yet be utilized [30], we plan instead to continue immediately towards a complete assessment of functional performance using a physical (real) multifunction prosthetic hand. The VR performance in this case study has been compelling and may suggest that simultaneous continuous control of a 3 DOF prosthetic hand is feasible. Following this, we will then attempt 4 DOF control using this same subject, both evaluating a few different controller approaches and controllable DOFs in VR once more.

Following all this, the elimination of the percutaneous interface through the use of a fully implanted recording system will be employed in the future. Such a system is presently in development and the current study participant could be upgraded without requiring removal or adjustment of the intramuscular electrodes, due to the standard connector between the percutaneous leads and the chronically implanted components. Future subjects are also expected to have an increased number of implanted electrodes (up to 16 total), which will increase the number of distinct signals and thus likely improve controller performance capability and the ability to control additional DOF. Lastly, a take home study will evaluate the actual functional impact on amputee activities of daily living.

### Long term impact

Future research can take advantage of intramuscular electrodes and incorporate such technology in the process of socket design or prosthetic hand development, as there would be fewer constraints if the placement of surface electrodes no longer needs to be taken into consideration. As direct placement of surface electrodes over muscles of interest would no longer be necessary, long-term comfort and stability could take precedence. Intramuscular electrodes could be used in tandem with approaches like osseointegration, or to allow for greater variation in choice of socket material. An intramuscular setup could also be useful for other levels of amputation such as shoulder disarticulation, particularly if Targeted Muscle Reinervation [42] approaches are utilized.

## Conclusion

Chronically implanted intramuscular electrodes offer considerable temporal and postural stability and provide a strong platform for advanced prosthetic controller algorithms. The utilization of such setups can reduce and potentially even eliminate the need for user retraining, offering a significant advantage over conventional surface electrode approaches. This opens up a variety of possibilities for new prosthetic designs and controller approaches.

## Data Availability

Data can be made available upon reasonable request.

## Abbreviations

ADC: Analog to Digital Converter
APL: Abductor Pollicis Longus
BW: Butterworth Filter
cm: Centimeter
DOF: Degree of Freedom
ECRB: Extensor Carpi Radialis Brevis
ECRL: Extensor Carpi Radialis Longus
EDC: Extensor Digitorum Communis
EDMP: Extensor Digiti Minimi Proprius
EMG: Electromyography (or electromyographic signals)
EPL: Extensor Pollicis Longus
FCR: Flexor Carpi Radialis
FCU: Flexor Carpi Ulnaris
FDA: Food and Drug Administration
FDP: Flexor Digitorum Profundus
FDS: Flexor Digitorum Superficialis
FES: Functional Electrical Stimulation
FPL: Flexor Pollicis Longus
g: Grams
Hz: Hertz
I/O: Input/Output
IDE: Investigational Device Exemption
IMES: Implantable Myoelectric Sensors
IM-MES: Intramuscular Myoelectric Electrode
IRB: Institutional Review Board
kHz: Kilohertz
KNN: K-Nearest Neighbor
MAV: Mean Absolute Value
MCP: Metacarpophalangeal
MES: Myoelectric signals
min: Minutes
MPL: The Modular Prosthetic Limb
MRI: Magnetic resonance imaging
ms: Milliseconds
NIP: Neural Interface Processor
OHMG: Osseointegrated Human-Machine Gateway
Pro T: Pronator Teres
s: Seconds
Sup: Supinator
VR: Virtual Reality
WFL: Waveform Length
